# A network analysis approach to ADHD symptoms: More than the sum of its parts

**DOI:** 10.1371/journal.pone.0211053

**Published:** 2019-01-18

**Authors:** Timothy J. Silk, Charles B. Malpas, Richard Beare, Daryl Efron, Vicki Anderson, Philip Hazell, Brad Jongeling, Jan M. Nicholson, Emma Sciberras

**Affiliations:** 1 School of Psychology, Deakin University, Geelong, Australia; 2 Murdoch Children’s Research Institute, Melbourne, Australia; 3 Department of Paediatrics, University of Melbourne, Melbourne, Australia; 4 Department of Medical Education, Melbourne Medical School, The University of Melbourne, Australia; 5 The Royal Children’s Hospital, Melbourne, Australia; 6 Discipline of Psychiatry, University of Sydney, Sydney, Australia; 7 Joondalup Child Development Centre, Perth, Australia; 8 Department of Paediatrics, University of Western Australia, Perth, Australia; 9 Judith Lumley Centre, La Trobe University, Melbourne, Australia; QIMR Berghofer Medical Research Institute, AUSTRALIA

## Abstract

In interpreting attention-deficit/hyperactivity disorder (ADHD) symptoms, categorical and dimensional approaches are commonly used. Both employ binary symptom counts which give equal weighting, with little attention to the combinations and relative contributions of individual symptoms. Alternatively, symptoms can be viewed as an interacting network, revealing the complex relationship between symptoms. Using a novel network modelling approach, this study explores the relationships between the 18 symptoms in the Diagnostic Statistical Manual (DSM-5) criteria and whether network measures are useful in predicting outcomes. Participants were from a community cohort, the Children’s Attention Project. DSM ADHD symptoms were recorded in a face-to-face structured parent interview for 146 medication naïve children with ADHD and 209 controls (aged 6–8 years). Analyses indicated that not all symptoms are equal. Frequencies of endorsement and configurations of symptoms varied, with certain symptoms playing a more important role within the ADHD symptom network. In total, 116,220 combinations of symptoms within a diagnosis of ADHD were identified, with 92% demonstrating a unique symptom configuration. Symptom association networks highlighted the relative importance of hyperactive/impulsive symptoms in the symptom network. In particular, the ‘motoric’-type symptoms as well as *interrupts* as a marker of impulsivity in the hyperactive domain, as well as *loses things* and *does not follow instructions* in the inattentive domain, had high measures of centrality. Centrality-measure weighted symptom counts showed significant association with clinical but not cognitive outcomes, however the relationships were not significantly stronger than symptom count alone. The finding may help to explain heterogeneity in the ADHD phenotype.

## Introduction

Attention-deficit/hyperactivity disorder (ADHD) is a common neurodevelopmental disorder with a worldwide prevalence in children of approximately 5% [1]. A hallmark of ADHD is its heterogeneity between individuals and within individuals over time in clinical presentations, cognitive profiles and comorbidities [2–5]. It is likely that this heterogeneity contributes to the documented variation in response to treatments. A more sophisticated understanding of within-syndrome symptom profiles may help progress towards a ‘precision medicine’ approach.

Diagnosis of ADHD is based on the presence/absence of 9 Inattentive and 9 Hyperactive/Impulsive symptoms according to the Diagnostic and Statistical Manual of Mental Disorders (DSM; DSM-IV and retained in DSM-5). Diagnostic subtypes defined in DSM-IV have been renamed ‘current presentations’ in DSM-5 partially in recognition of their instability over time [2,6]. Three presentations are: ‘predominantly inattentive’ for the presence of 6 or more Inattentive and fewer than 6 Hyperactive/Impulsive symptoms, ‘predominantly hyperactive/impulsive’ for 6 or more Hyperactive/Impulsive symptoms and fewer than 6 Inattentive symptoms, or ‘combined’ for 6 or more symptoms in both domains. If these criteria are applied strictly, merely comparing individuals or groups categorically may mask subtle phenotypic variations. For example, a child with 6 inattentive symptoms is diagnosed with ‘ADHD’ whereas another child with 5 inattentive symptoms is considered ‘healthy’. In contrast, research relies more on a dimensional approach to assess psychopathology on a continuum, and typically examines the number of symptoms to indicate severity (i.e. 9 symptoms are worse than 6, which are worse than 3). Although there are advantages and disadvantages of the two approaches, both rely on a binary symptom count (present or absent) with individual symptoms having equal weighting. Little attention is given to individual symptom profiles, therefore two individuals with same clinical diagnosis and symptom counts may have quite different profiles and clinical presentations. By focussing only on the sum of symptoms, useful clinical information may be lost, hampering efforts to determine underlying aetiological factors and potential biomarkers. An alternative novel approach is to view ADHD as a network of interacting symptoms, providing detailed information on the complex relations between symptoms. While all 18 of the DSM symptoms contribute significantly and independently to the disorder [7], given the heterogeneity of the ADHD presentation, identifying symptoms that are central to the disorder have multiple implications including better understanding aetiological factors associated with the disorder, increasing accuracy in predicting outcomes and informing treatment approaches.

This concept of a network of interacting symptoms is reviewed in Borsboom and Cramer [8]. Borsboom and colleagues have used a network analysis approach to conceptually understand psychopathology across the entire DSM [9]. A series of studies has subsequently demonstrated the benefit of examining relationships of individual symptoms in mood disorders, leading to the identification of different risk factors, gene polymorphisms, psychosocial functioning, and patterns of comorbidity [10–14]. Recently, Martel *et al*. [15] used this approach in ADHD symptomatology, examining the symptom network across four developmental age groups (3-6yrs, 6-12yrs, 13-17yrs and 18-36yrs). This study demonstrated that symptom clusters become more differentiated and less clustered over development. However across development two symptoms, *Often easily distracted* and *Difficulty sustaining attention* seem to be central symptoms across the age ranges. Extending Martel *et al*. [15], the current study examines the networks separately for ADHD and non-ADHD controls and examines whether information on the centrality of symptoms can be used to predict concurrent or future clinical and cognitive presentations.

This study aimed to 1) explore the structure of the symptomology network in children with and without ADHD, using the 18 ADHD symptoms in the DSM; 2) use centrality and cluster coefficients to capture the complexity and topology of the symptom network, and 3) to examine whether information on the centrality of symptoms can be used to predict concurrent or future clinical and cognitive presentations.

## Materials and methods

### Participants

This paper reports on a subsample of the baseline cohort from the Children’s Attention Project (CAP), a longitudinal community study of children with ADHD and non-ADHD controls. The CAP protocol and cohort has been described previously [16]. Briefly, participants were recruited in a two-stage screening and case-confirmation procedure.

Of 5922 eligible Grade 1 participants from 43 Melbourne primary schools, 3734 returned both parent and teacher screening reports on the Conners 3 ADHD Index [17]. Children screened positive as potential ADHD cases if their scores on *both* the parent and teacher ADHD indices were ≥75th percentile for age for boys, and ≥80th percentile for girls. In addition, any child reported by parents to have previously been diagnosed with ADHD was regarded as a positive screen. Children were classified as screening negative if their scores on *both* parent and teacher ADHD indices were <75th percentile for boys and <80th percentile for girls. A higher cut-point was used for girls based on our pilot data, which showed that this resulted in better correspondence with diagnostic confirmation. Of those eligible children with complete screening data, 412 children screened positive for ADHD and were matched (by sex and school) to 412 children screening negative. To confirm diagnostic status, a full parent face-to-face structured diagnostic interview was conducted (NIMH Diagnostic Interview Schedule for Children IV–DISC-IV [18]), completed by staff who have at least a four-year accredited undergraduate degree in psychology, were comprehensively trained in all procedures, and were blind to child screening status. Of those consented to participate in the longitudinal study, 179 children met criteria for ADHD, and of the matched negative screens, 212 were confirmed non-ADHD controls. All children were aged between 6–8 years. The subsample of participants included in the present study included only medication naïve children with ADHD (N = 146), and 209 non-ADHD controls (three were excluded for having 6 or greater than 6 of either inattentive or hyperactive/impulsive symptoms, despite not having the level of impairment to meet ADHD diagnosis). See Table 1 for sample characteristics. The ADHD group comprised 61 Predominantly Inattentive (ADHD-I), 15 Predominantly Hyperactive/Impulsive (ADHD-H) and 70 Combined type (ADHD-CT). For simplicity, further reference to these presentations will be refereed to as ‘subtypes’. The DSM-IV criteria for ADHD, derived from the DISC-IV diagnostic interview with the parent was used for subsequent analyses. Comorbidities of internalizing and externalizing disorders were assessed using the DISC-IV [18]. Children were classified as having an internalizing disorder if they met criteria for separation anxiety disorder, social phobia, generalized anxiety disorder, post-traumatic stress disorder, obsessive-compulsive disorder, hypomania or manic episode, and an externalizing disorder if they met criteria for oppositional defiant disorder or conduct disorder. Other sample characteristics in include the estimated full scale IQ, assessed using the vocabulary and matrix reasoning subtests of the Wechsler Abbreviated Scale of Intelligence (WASI)[19], and neighborhood socioeconomic disadvantage (Socio-Economic Indexes for Areas Disadvantage Index (SEIFA) [20])

**Table 1:** Sample characteristics for children with ADHD and non-ADHD controls.

	ADHD *n = 146*^#^	Control *n = 209*^#^	T/χ^2^	*p* *value*
*Child characteristics*				
Child age in years, mean (SD)	7.3 (0.4)	7.3 (0.4)	1.405	0.161
Male, n (%)	100 (68.5)	132 (63.2)	1.081	0.299
ADHD subtype n (%)				
ADHD-Combined	70 (47.9)			
ADHD-Inattentive	61 (41.8)			
ADHD-Hyperactive/Impulsive	15 (10.3)			
Internalizing disorder,^a^ n (%)	37 (25.3)	10 (4.8)	31.625	<0.001
Externalizing disorder^a^, n (%)	75 (51.4)	16 (7.7)	86.165	<0.001
Estimated full scale IQ standard score,^b^ mean (SD)^#^	92.5 (12.1)	101.4 (13.5)	6.329	<0.001
SEIFA,^c^ mean (SD)^#^	1014.9 (41.1)	1015.6 (45.6)	0.160	0.873

^a^ DISC-IV

^b^ Wechsler Abbreviated Scales of Intelligence

^c^ Socio Economic Indexes for Areas Disadvantage

^d^DISC-IV

^#^ *n* for SEIFA, ADHD = 143, Controls = 207; *n* for IQ, ADHD = 144.

### Ethical considerations

Parents provided consent for participation in each stage of the study. Approval was obtained from the Human Research Ethics Committees of The Royal Children’s Hospital (#31056) and the Victorian Department of Education and Early Childhood Development (#2011_001095).

### Diagnostic criteria

Presence or absence of symptoms was determined using the ADHD module from the DISC-IV diagnostic interview with the parent. The DSM-IV criteria for ADHD (retained in DSM-5) are listed below, followed in parentheses by the contraction used for further description and the abbreviation used in figures. *Inattentive criteria*: (1) Often fails to give close attention to details or makes careless mistakes in schoolwork, work, or other activities (*fails close attention* [closeatt]); (2) Often has difficulty sustaining attention in tasks or play activities (*difficulty sustaining attention* [susatt]); (3) Often does not seem to listen when spoken to directly (*does not listen* [listen]); (4) Often does not follow through on instructions and fails to finish schoolwork, chores, or duties in the workplace (*does not follow instructions* [instruct]); (5) Often has difficulty organizing tasks and activities (*difficulty organizing* [org]); (6) Often avoids, dislikes, or is reluctant to engage in tasks that require sustained mental effort (*avoids mental effort* [avoid]); (7) Often loses things necessary for tasks or activities (*loses things* [loses]); (8) Is often easily distracted by extraneous stimuli (*easily distracted* [distract]); (9) Is often forgetful in daily activities (*forgetful* [forget]).

*Hyperactive/ Impulsive criteria*: (1) Often fidgets with hands or feet or squirms in seat (*fidgets* [fidget]); (2) Often leaves seat in classroom or in other situations in which remaining seated is expected (*leaves seat* [seat]); (3) Often runs about or climbs excessively in situations in which it is inappropriate (*runs excessively* [runs]); (4) Often has difficulty playing or engaging in leisure activities quietly (*difficulty playing quietly* [quiet]); (5) Is often "on the go" or often acts as if "driven by a motor" (*driven by motor* [motor]); (6) Often talks excessively (*talks* [talks]); (7) Often blurts out answers before questions have been completed (*blurts* [blurts]); (8) Often has difficulty awaiting turn (*awaiting turn* [turn]); (9) Often interrupts or intrudes on others (*interrupts* [interrupt]).

### Configural frequency

In an approach to understand the heterogeneity of the ADHD symptoms, we first examined the symptom profile configuration. For the number of combinations for any given symptom count, the following binomial coefficient formula was used [21], whereby *n* is the number of potential symptoms and *k* is the number of the subset:

(nk)=n!(n−k)!k!

Therefore, if an individual has 7/9 inattentive symptoms (n/k), there are 36 combinations of inattentive symptoms. However, for a diagnosis of ADHD-I an individual with 7 inattentive symptoms may still have 0–5 hyperactive/impulsive symptoms. For 7 inattentive symptoms and one hyperactive/impulsive symptom, there are 324 (36 x 9) possible combinations. The number of configurations was calculated across all symptom count combinations.

### Outcome measures

Outcome measure were a range of clinical and academic cognitive functioning measures assessed at the time of the diagnosis and/or as part of a 3 year follow up (See S1 Table). Comorbidities of internalizing and externalizing disorders were assessed as described above. Irritability was assessed using the parent-reported Affective Reactivity Index [22], and social problems were assessed using the parent-reported peer problems subscale from the Strengths and Difficulties Questionnaire (SDQ [23]). Quality of life measures were derived using subscales from the Child Health Questionnaire (CHQ [24]): family activities (e.g., how often has your child’s behavior interrupted various everyday family activities [eating meals, watching TV]); time impact (e.g., how often has your child’s behavior caused you to cancel or change plans [personal or work] at the last minute); and emotional impact (e.g., how much worry or concern did the child’s emotional well-being or behavior cause you). Academic cognitive functioning was assessed using the Word Reading and Math Computation subtests from the Wide Range Achievement Test 4 (WRAT 4 [25]), and the Clinical Evaluation of Language Fundamentals (fourth edition; CELF-4) screening test [26], We also collected a teacher-reported 7 item measure of academic competence from the Social Skills Improvement System (SSIS [27]).

### Statistical analyses

All statistical analyses were performed in *R* (r-project.org). Following McNally *et al*. [28] symptom networks were constructed using the *qgraph* package [29].

#### Association and concentration networks

As a first step, a correlation matrix was computed representing the zero order correlations between symptoms. This was conducted on the ADHD and control groups separately, as well as on the entire samples. As symptoms were coded in a binary fashion, this matrix contained phi coefficients for each symptom pair. A weighted network representation of this matrix was constructed using *qgraph*. The network nodes (circles) represent the DSM-5 ADHD symptoms, with the edges (connections between nodes) representing the phi coefficients or the association between symptoms. The Fruchterman Reingold algorithm [30] was used to visualize the network. This algorithm reorganizes the nodes of the network such that nodes with stronger intercorrelations are positioned proximal to one another, while nodes with weaker connections are positioned more distally. This allows clusters of related nodes to be identified visually. No connection threshold was applied to this first network in order to fully visualize the range of intercorrelations. To limit potentially spurious connections resulting from stochastic error, a second network was computed following a similar process, but with connections thresholded based on Family-wise error (FEW) *p* < .05, using Hochberg's procedure [31]. For 80% power at alpha = .05 (two-tailed) this analysis was powered to detect medium to large effect sizes (r > .3).

#### Centrality and clustering coefficient

Graph theory metrics were computed to capture the complexity and topology of the symptom network. Centrality metrics describe the relative importance of individual nodes within the network, while clustering metrics capture the relationship between each node and its neighbours. Three centrality metrics were computed: betweenness, closeness, and strength [32], whereby higher values indicate higher relative importance within the network. The *betweenness* for a node represents the number of shortest paths between all nodes in the graph that pass through that node. Nodes with high betweenness are highly connected to other nodes and occupy the position of ‘hubs’ within the network. Nodes with high betweenness are likely to be “gateway” nodes–for example a town on a major highway may not be directly connected to many other towns, but would have a high betweenness score because the highway is one link in the shortest paths between many towns. *Closeness* is the inverse of the average path length between a node and all other nodes in the graph. As such, nodes with higher *closeness* have high average correlations with all other nodes in the graph. Both of these metrics depend on the computation of the shortest path length between nodes. In a weighted graph, the shortest path length is defined as the path of least resistance using the approach described by Dijkstra [33]. The *strength* of a node is the sum of weights of edges connected to the node. In this case weights are correlation coefficients. For the *clustering* coefficient we used the approach for weighted networks described by Zhang and Horvath [34]. Briefly, nodes with high clustering coefficients are connected to neighbours that are highly connected with each other.

#### Outcome prediction

To test whether information on the centrality of symptoms improved the outcome prediction in ADHD, node strength and clustering coefficient were used to predict clinical outcomes following the approach by Boschloo et al. [35]. First, the number of endorsed symptoms was summed to form an unweighted symptom scores. Next, a weighted symptom score was formed by multiplying each symptom by the corresponding node strength and summing the weighted values. This procedure was repeated for clustering coefficients. Kendall’s [36] rank correlation coefficient (tau) was computed between symptom scores and continuous outcome variables. Mean differences between binary outcome variables were examined using Welch’s [37] unequal variances t-test. Differences between correlation coefficients were examined using the non-parametric bootstrap (2,000 samples) with associated 95% bias corrected and accelerated confidence (BCa) intervals.

## Results

### Participant characteristics

The ADHD and control groups did not differ in age, sex distribution, or socioeconomic disadvantage. As expected, the ADHD group presented with more internalizing and externalizing disorder comorbidity, and had lower IQ than the control group (See Table 1).

### Frequency of specific symptom endorsement

**Fig 1:**
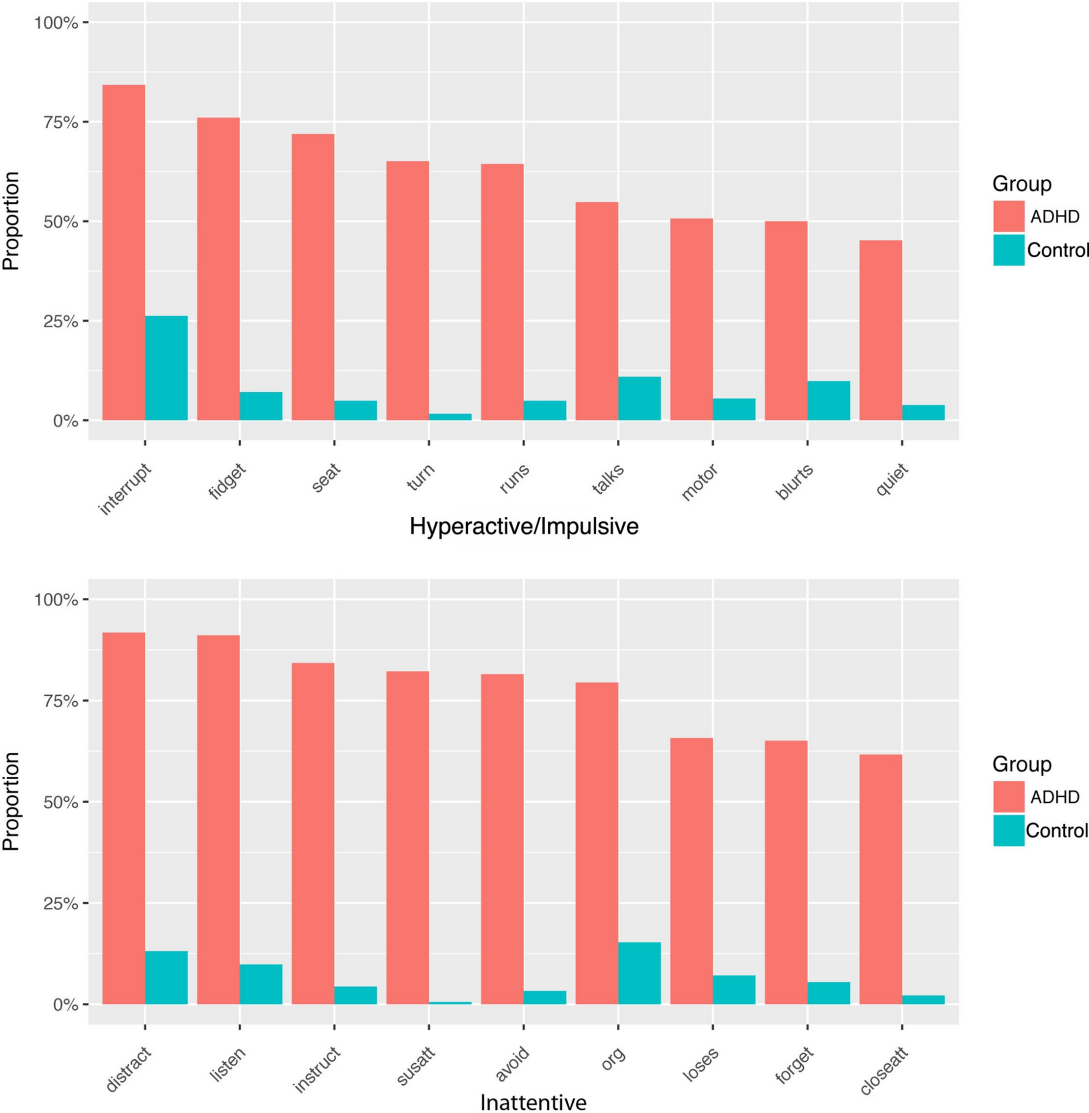
Hyperactive/Impulsive (above) and Inattentive (below) symptom endorsement frequency for the ADHD participants and non-ADHD controls, ordered in descending frequency of presentation within the ADHD group.

In Fig 1, each diagnostic criterion is listed as a percentage of symptom endorsement for children with ADHD and non-ADHD controls. Of the inattentive symptoms, *‘easily distracted’* and ‘*does not listen’* were the most frequently endorsed symptoms in the ADHD group overall, as well as in each of the subtype categories (*difficulty sustaining attention* was equal second for the hyperactive/impulsive subtype). Even for the non-ADHD group, while being much less frequent, these two symptoms were the second and third most frequently endorsed, behind *‘difficultly organizing’*.

For the hyperactive/impulsive symptoms, *‘interrupts’* was the most frequently endorsed symptom across the ADHD group and each of the subgroups, as well as for the non-ADHD group. For ADHD-H and ADHD-CT this symptom was endorsed for 100% of participants. The symptom endorsement frequencies for the ADHD subgroups are presented in S1 Fig.

### Count of symptom endorsement

Table 2 shows the summed count on symptom endorsement for each ADHD subtype and non-ADHD controls. Although the ADHD-I group required ≥ 6 inattentive and < 6 hyperactive/impulsive symptoms, almost half of the ADHD-I group were just 1 or 2 hyperactive/impulsive symptoms short of being classified as ADHD-CT. Similarly, of the ADHD-H group, two thirds were only 1 or 2 inattentive symptom short of being classified as ADHD-CT. Of the ADHD-CT subgroup 21.4 and 30.0% had only 6 symptoms endorsed for inattentive and hyperactive/impulsive symptoms respectively. If they had one fewer symptom in either category, they would fit one of the ADHD-H or ADHD-I subtypes.

**Table 2:** Summed symptom count for each subtype.

	Inattentive symptoms			Hyperactive/Impulsive symptoms		
	Symptom count	Frequency (n)	%	Symptom count	Frequency (n)	%
**Inattentive subtype (n = 61)**						
	6	21	34.4	0	6	9.8
	7	19	31.1	1	4	6.6
	8	13	21.3	2	8	13.1
	9	8	13.1	3	13	21.3
				4	9	14.8
				5	21	34.4
**Hyperactive/Impulsive subtype (n = 15)**						
	0	0	0	6	7	46.7
	1	1	6.7	7	4	26.7
	2	3	20	8	2	13.3
	3	1	6.7	9	2	13.3
	4	4	26.7			
	5	6	40			
**Combined type (n = 70)**						
	6	15	21.4	6	21	30
	7	13	18.6	7	16	22.9
	8	24	34.3	8	18	25.7
	9	18	25.7	9	15	21.4
**Controls (N = 209)**						
	0	127	60.8	0	113	54.1
	1	34	16.3	1	38	18.2
	2	23	11.0	2	30	14.3
	3	7	3.3	3	13	6.2
	4	13	6.2	4	5	2.4
	5	5	2.4	5	10	4.8

### Configural frequency

To understand the heterogeneity of the ADHD symptoms, we first examined the symptom profile configuration of all participants with ADHD. Although classification can be divided into inattentive, hyperactive/impulsive or combined subtype, when we assess the unique configuration of the 18 individual symptoms there were 116,220 possible combinations of individual symptom criteria that individuals could have to qualify for a diagnosis of ADHD; 49,660 configurations for each of ADHD-I and ADHD-H subtypes, and 16,900 configurations for ADHD-CT. When examining the configural frequency within the ADHD group, there was considerable symptom variability, with 91.8% (N = 134) of individuals having a unique symptom pattern. Endorsement of all 18 symptoms was the most common pattern, shared by eight ADHD individuals. There were a further two patterns that were shared by two participants each (each with 17 symptoms).

### Describing symptom networks

**Fig 2:**
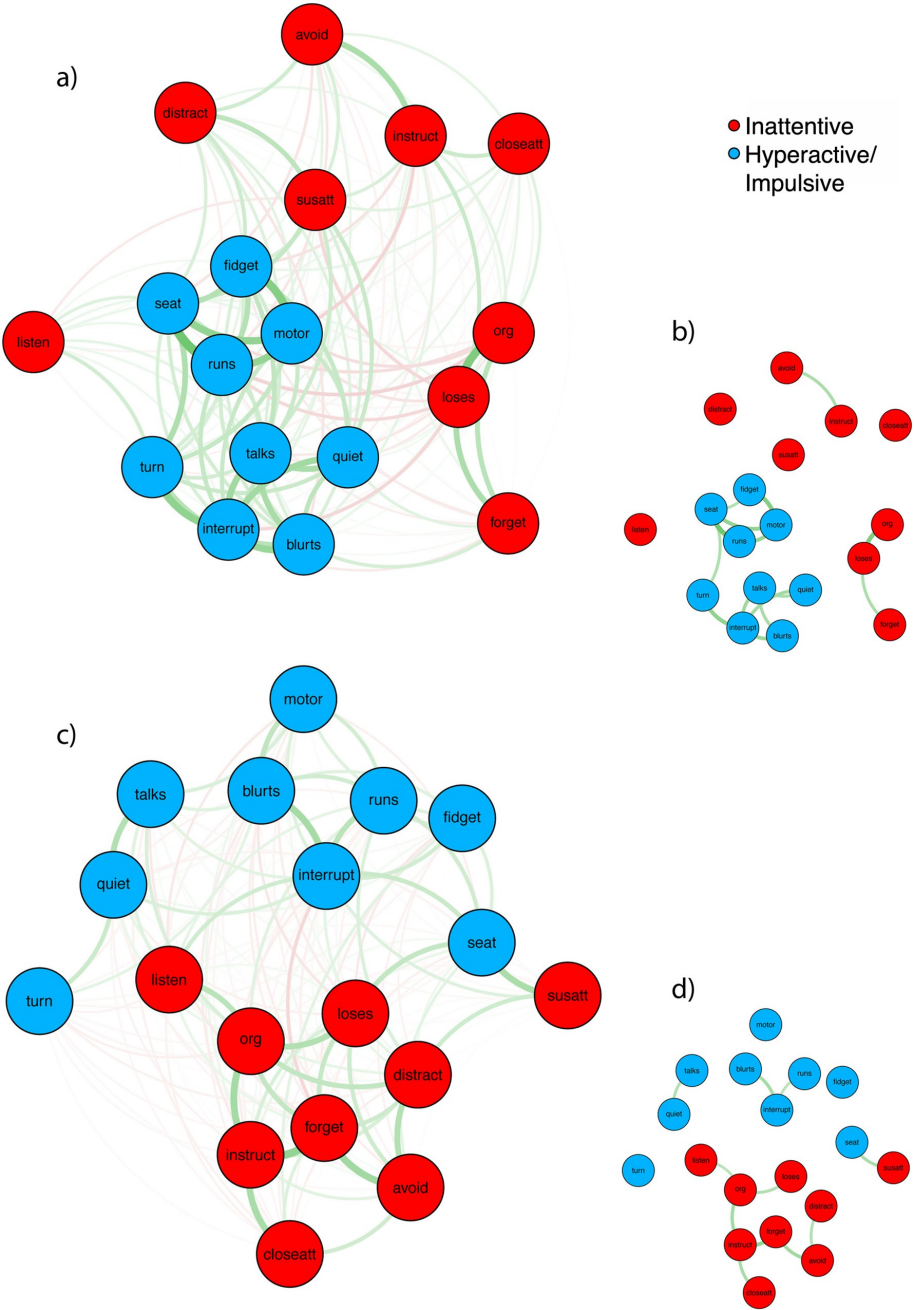
The association network for (a) all ADHD participants, and (b) thresholded with only edges that survive FWE p<0.05; (c) for all non-ADHD control participants,and (d) thresholded with only edges that survive FWE p<0.05. Nodes represent each of the 18 ADHD symptom criteria, connected by edges characterizing the zero-order Phi correlations between symptoms. Inattentive symptoms are presented in red and the hyperactive/impulsive symptoms are presented in blue. The thickness of the edges represents the magnitude of the association. Positive correlations were displayed as green and negative correlations were displayed as red.

Fig 2 presents the association networks separately for ADHD and non-ADHD controls, and the association network for the combined sample is supplied in S2 Fig. Fig 2A shows the symptom network for all participants with ADHD. Overall, symptoms separated well into inattentive and hyperactive/impulsive domains. Visual inspection reveals hyperactive/impulsive symptoms were more closely clustered than the inattentive symptoms, and were organized into two smaller groupings: ‘motoric’-type symptoms (*leaves seat*, *runs excessively*, *fidgets*, *driven by motor*), and ‘inhibition’-type symptoms (*interrupts*, *blurts*, *talks*, *difficulty playing quietly*). These two groupings are bridged by difficulty ‘*awaiting turn*’.

Inattentive symptoms were more dispersed, but also revealed two general clusters, with strong association among symptoms of planning/organization (*difficulty organizing*, *loses things*, *forgetful*) and more loosely symptoms of sustaining attention (*easily distracted*, *difficulty sustain attention*, *fails close attention*, *avoids mental effort* and *does not follow instructions*). Out of the inattentive symptoms *does not listen* was mostly isolated.

This pattern became more pronounced when the network was thresholded at *p* < .05 (Fig 2B). The clustering of the hyperactive symptoms was preserved, including the ‘motoric’ and ‘inhibition’ sub-clusters. Only three edges in the inattentive symptom group survived thresholding. These were between *avoid mental effort* and *does not follow instructions*, and between *loses things* and *difficulty organising things* as well as *forgetful*.

The association network map for the non-ADHD group is presented in Fig 2C for all associations and Fig 2D after thresholding. This network similarly separates inattentive and hyperactive symptoms, however hyperactive symptoms are less tightly clustered and inattentive symptoms have higher association with each other. Centrality measures highlight *interrupts* and *difficulty organising* as the most important to the symptom network for non-ADHD controls.

### Centrality and clustering coefficient

**Fig 3:**
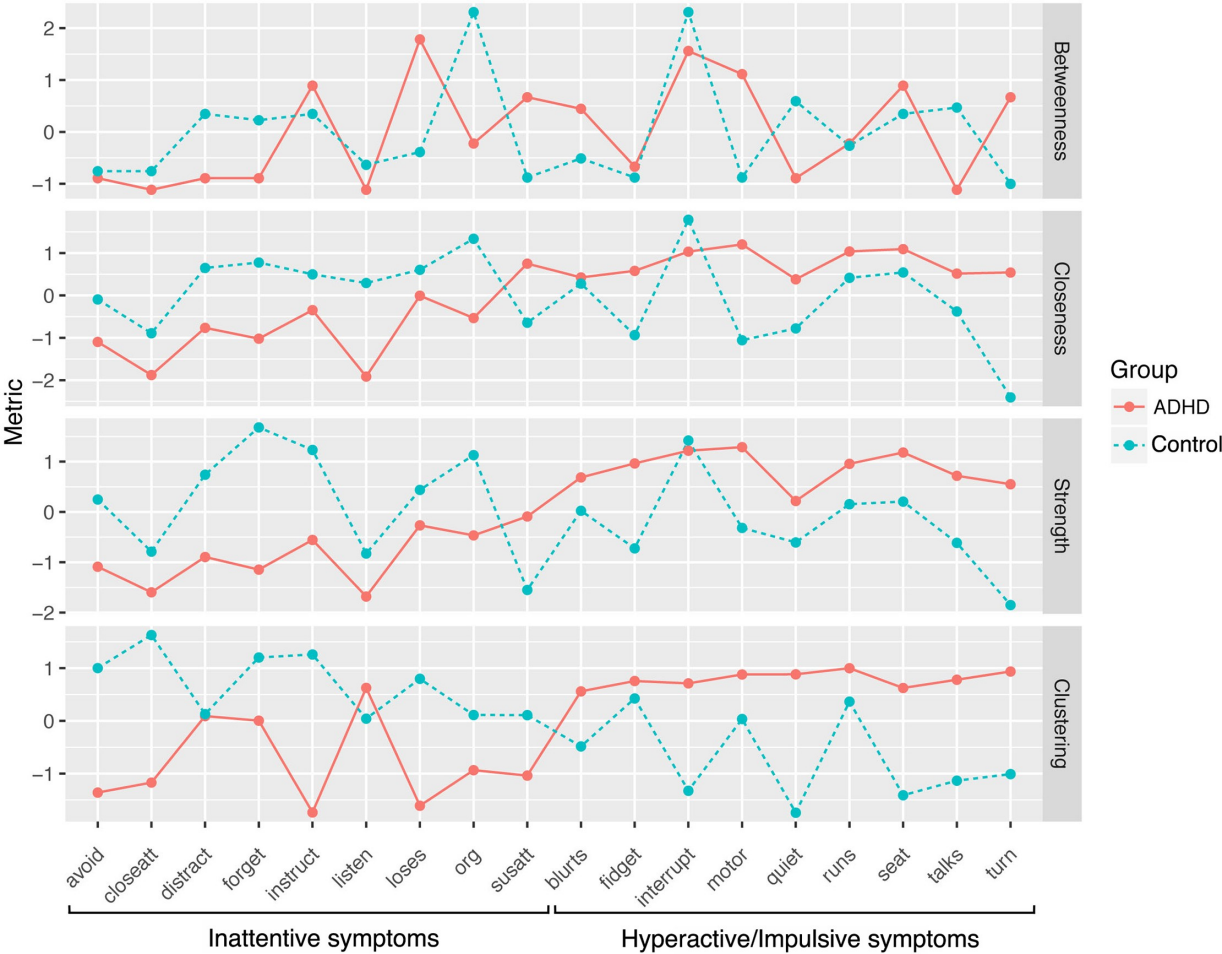
Centrality and clustering metrics plotted for each symptom for the ADHD participants and the non-ADHD controls. Plots are presented for betweenness centrality, closeness centrality, node strength and clustering coefficients. Symptoms are ordered alphabetically (left to right) within inattentive symptoms followed by hyperactive/impulsive symptoms.

Measures of centrality, derived from the network analysis, calculate how connected individual symptoms are, with higher values reflecting greater relative importance of the symptom in the network. These are graphed in Fig 3. Greater betweenness centrality for a symptom indicates that it tends to lie along the shortest path connecting other symptoms. For participants with ADHD, ‘*loses things’* from the inattentive domain, was the node with the highest betweenness centrality. Although this symptom appears to be in the periphery of the network as represented in Fig 2, it has strong correlation between the neighbouring nodes. The shortest paths connecting these nodes are strong, contributing to the weighted betweenness centrality metric. This is supported by the relatively lower closeness centrality and node strength, indicating the connectivity to all other nodes is relatively low. For participants with ADHD, the next three symptoms with greatest betweenness centrality are from the hyperactive domain, with *interrupts*, *driven by motor* and *leaves seat* having short path lengths to their neighbouring symptoms. This is consistent with their position in the central component of the association network in Fig 2.

Closeness centrality, node strength and clustering coefficients showed a very similar pattern to each other, confirming the separation of hyperactive and inattentive symptoms (Fig 2). With the exception of *difficulty sustaining attention* in closeness centrality and *listens* in clustering, across the three metrics the hyperactive/impulsive symptoms are ranked higher than all the inattentive symptoms in relative importance to the network. For closeness centrality and node strength, the ‘motoric’-type symptoms (*leaves seat*, *runs excessively*, *fidgets*, *driven by motor*) and *interrupts*, ranked the highest five hyperactive/impulsive symptoms. Nodes from the hyperactive/impulsive cluster also had very high clustering coefficients, indicating that their immediate neighbours are highly connected to each other. In contrast, the clustering coefficient was generally lower for the inattentive symptoms. *Does not listen* and *fails close attention* from the inattentive domain were the least central nodes by all measures of centrality. Although *does not listen* had low centrality, its clustering coefficient was high. This is most likely explained by its proximity to both the motoric and inhibition sub-clusters of the hyperactive symptom domain that are in term both highly clustered, as seen in Fig 2.

Overall, these analyses highlight the importance of the hyperactive symptoms in the symptom network. In particular the ‘motoric’-type symptoms and *interrupts*, appeared to be the most significant.

### Outcome prediction

As seen in Table 3 the unweighted, strength-weighted, and clustering-weighted symptom counts were associated with each clinical measure for individuals with ADHD. All symptom count measures were greater in participants with either internalizing or externalizing comorbidities. All symptom counts predicted irritability and quality of life (across each domain; emotional, family, and time) at the 3 year follow-up, and parent reported social problems were predicted at both time points. None of the symptom counts predicted any of the academic cognitive functioning measures either at baseline or at follow-up. The weighted symptom counts showed the biggest difference in predicting internalizing or externalizing comorbidities, however there was no statistical evidence for stronger relationships with the weighted, versus unweighted, symptom counts.

**Table 3:** Relationship between total symptom count and outcomes.

*Outcome*			Unweighted		Weighted (Strength)		Weighted (Clustering)	
	Baseline	3yr	Stat.	Sig.	Stat.	Sig.	Stat.	Sig.
*Clinical measures*								
Externalising comorbidity*	•		-0.48 [-0.80, -0.14]	**.005**	-0.56 [-0.89, -0.22]	**.001**	-0.53 [-0.86, -0.20]	**.002**
Internalising comorbidity*	•		-0.49 [-0.88, -0.11]	**.011**	-0.54 [-0.94, -0.17]	**.004**	-0.53 [-0.93, -0.16]	**.006**
Irritibility		•	.17 [.04, .30]	**.018**	.17 [.04, .30]	**.013**	.17 [.04, .29]	**.015**
Social problems	•		.13 [.04, .23]	**.03**	.13 [.04, .23]	**.026**	.12 [.03, .22]	**.036**
		•	.22 [.10, .33]	**.002**	.19 [.08, .31]	**.004**	.20 [.09, .32]	**.003**
QoL emotional		•	-.18 [-.30, -.07]	**.011**	-.17 [-.29, -.05]	**.013**	-.17 [-.29, -.05]	**.012**
QoL family		•	-.15 [-.29, -.02]	**.026**	-.15 [-.28, -.02]	**.024**	-.15 [-.28, -.01]	**.028**
QoL time		•	-.19 [-.31, -.08]	**.011**	-.19 [-.30, -.07]	**.010**	-.18 [-.29, -.06]	**.013**
*Academic cognitive functioning*								
WRAT maths	•		-.02 [-.12, .08]	.76	.00 [-.10, .11]	.95	.00 [-.11, .10]	.95
		•	.01 [-.12, .14]	.84	.01 [-.12, .14]	.85	.01 [-.12, .14]	.88
WRAT reading	•		.03 [-.08, .14]	.57	.03 [-.08, .14]	.57	.03 [-.08, .14]	.59
		•	.02 [-.11, .16]	.74	.01 [-.12, .15]	.85	.01 [-.12, .15]	.85
CELF language	•		.00 [-.09, .09]	.98	.00 [-.10, .07]	.81	-.01 [-.10, .08]	.92
		•	-.01 [-.10, .09]	.93	-.03 [-.13, .06]	.66	-.01 [-.11, .08]	.85
Academic competence	•		.00 [-.12, .11]	.94	.01 [-.10, .12]	.90	.01 [-.10, .12]	.91
		•	.06 [-.08, .20]	.43	.07 [-.08, .21]	.33	.06 [-.08, .21]	.36

**Note:** statistic for continuous outcomes is Kendall’s rank correlation coefficient (tau) with 95% CIs.

Statistic for dichotomous outcomes (indicated with *) are Cohen’s *d* with 95% CIs and *p* values derived from Welch’s unequal variances t-test. Bold significance values indicate *p* < .05 (two-tailed).

## Discussion

This paper presents a novel approach for modeling ADHD symptom profiles, which takes into account the patterns of association between symptoms. We found that not all symptoms were equal, having different frequencies of endorsement, and different configurations of symptoms, with certain symptoms playing a more important role within the ADHD symptom network.

Few studies have examined the frequency of ADHD symptom endorsement, however the pattern of endorsement is remarkably similar to Canadian [38] and US [39] community samples in pre- and early school years. In the current study, *‘easily distracted’* and ‘*does not listen’* were two of the most frequently endorsed inattentive symptoms in both the ADHD (and each subtype) and control groups. In a large review and meta-analysis conducted to evaluate the validity of the DSM-IV framework of ADHD and inform decision-making for DSM-5, Willcutt *et al*. [2] noted that the high prevalence of these two symptom criteria in controls results in their having weak utility for categorically discriminating ADHD from healthy controls. Factor analyses found ‘*does not listen’* cross-loads heavily on the hyperactive/impulsive factor. This is also supported by the network analysis, which shows ‘*does not listen*’ isolated from the other inattentive symptoms and in close proximity to the hyperactive/impulsive cluster. While the issue of symptom utility is important for a clinical distinction, such symptoms may still be useful in examining the underlying and perhaps causal nature of interacting symptoms. Relatively high clustering coefficient of these two symptoms demonstrates that they co-occur with highly associated clusters however both have low importance within the network. This may mean that they are a result of the presence of other key symptoms.

On the other hand, *‘interrupts’* was the most frequently endorsed hyperactive/impulsive symptom across all groups (each ADHD subtype and non-ADHD controls) and one of the highest ranked symptoms across network measures, supporting the importance of this symptom within the symptom network. In fact, if a non-ADHD participant had *interrupts* as an endorsed symptom then they had a mean symptom count of four. If not, the mean symptom count was less than one. This high prevalence and importance in the network could suggest that it is a sensitive marker of hyperactivity/impulsivity and potentially may have a downstream causal relationship to the development of other symptoms. If future longitudinal studies reveal a causal relationship such symptoms could offer potential predictors of onset or a target for intervention.

Exploratory and confirmatory factor analyses consistently support the separation of inattentive and hyperactive/impulsive symptoms [2]. Our network analysis supports this distinction, in particular the high association within hyperactive/impulsive symptoms. However, in examining the symptom profile, based on the summation of the symptom criteria, a large proportion of participants within each subgroup are only one or two symptoms away from an alternative subgroup classification. This is consistent with the poor test-retest reliability estimates for the subtypes and the propensity for instability of diagnostic subtypes over time [6], and a key reason for the change in nomenclature from subtype to ‘current presentation’ in the DSM-5. It is also easy to see why there are inconsistencies reported in between-subtypes studies in, for example, the cognitive profile or structural and functional neuroimaging.

In examining the symptom association network, the findings are remarkably similar to that of the child group in a US cohort [15]. Martel *et al*. [15] presents the symptom network across a sample of ADHD and controls combined (comparable to S2 Fig). Both the US cohort and the current study, across ADHD and controls combined, find good segregation of inattentive and hyperactive/impulsive symptoms, with slightly higher clustering from inattentive symptoms, and similar grouping of individual symptoms. Extending Martel *et al*.[15], the current study also examines the networks separately for ADHD and non-ADHD controls, showing notable differences. When examined separately, both the association networks and centrality measures show that in healthy controls, the inattentive symptoms are more tightly and important within the network (with the exception of ‘*interrupts*’). This does not suggest that inattention symptoms are more affected in controls, but rather proportionally within the control network, certain inattention symptoms are likely to co-occur more commonly than other symptoms. This would be expected given that symptoms of inattention can occur in many other condition, as well as more commonly present in the normal population without clinical impairment. For individuals with ADHD, the hyperactive/impulsive symptoms are more tightly clustered highlighting the importance of hyperactive/impulsive symptoms in the symptom network.

Symptoms varied considerably in their centrality and clustering within the network, which implies that certain symptoms may carry particular clinical and/or neurobiological significance. The ‘motoric’-type symptoms and *interrupts* in the hyperactive/impulsive domain, and *loses things* and *does not follow instructions* in the inattentive domain, held high importance in the network. In examining whether information on the centrality of symptoms improves outcome predictions compared to symptom severity (count) alone, both strength-weighted and clustering-weighted symptom counts were assessed. Both weighted symptom measures demonstrated significant association with all clinical measures: internalizing and externalizing comorbidity, irritability, social problems and quality of life, but not for any measure of academic cognitive functioning. While some of the weighted symptom counts showed a better prediction of some clinical measures, particularly the presence of internalizing or externalizing comorbidities, the relationships were not significantly stronger than symptom count alone.

### Limitations

A number of limitations of the research need to be acknowledged. Inherent to behaviorally-defined disorders in children, the symptom endorsement is reliant on a third party, in which there are certain biases and errors. Further replication is required using different informants (e.g. teachers, self-report). Secondly, although an advantage of this cohort is the narrow age band (6-8years), restricting age-associated variance, it also limits the generalizabilty. The symptom network may indeed look different at different stages of development. The cohort in this study is being followed up longitudinally and further work will examine whether/how the network changes. Despite the emerging and promising framework of the network approaches in psychiatry there are also pitfalls that should be kept in mind [40–42]

### Future research

Conceptualizing psychiatric symptoms as a network of interacting symptoms offers an alternative to traditional categorical and dimensional approaches, and opens up a wealth of future questions to explore. Are there symptoms or combinations of symptoms that are worse to have than others? Does the pattern of individual symptoms change over development? Do the presence of certain symptoms or clusters change with diagnostic persistent versus remission? Are certain symptoms affected by different genotypes, and do they have different implications for brain structure and function? Answers to such questions will reveal clinically useful information and provide greater insight into the heterogeneity of ADHD. Such utility could be ascertained with longitudinal analysis of symptom networks and the combination of neuroimaging and genetics.

This study provides a unique approach in examining the network structure of ADHD symptoms. Moving beyond classical categorical and dimensional approaches, conceptualizing the symptoms as a network of interacting features has the potential to reveal symptoms of clinical importance. The current findings provide a platform for exploring the relationship between symptom clusters and a range of outcomes relevant to the understanding and management of ADHD.

## Supporting information

S1 Fig The symptom endorsement frequency for each of the ADHD subtypes. (TIF)

S2 Fig The association network for all participants (ADHD and non-ADHD controls). Nodes represent each of the 18 ADHD symptom criteria, connected by edges characterizing the zero-order Phi correlations between symptoms. Inattentive symptoms are presented in red and the hyperactive/impulsive symptoms are presented in blue. The green edges represent positive correlations and the thickness of the lines represent the magnitude of the association.
(TIF)

S1 Table Clinical and academic outcomes for children with ADHD. (DOCX)

S1 Dataset Dataset. (CSV)
