# Supplementary figures and images for: A network analysis approach to ADHD symptoms: More than the sum of its parts
Source: PLoS One. 2019 Jan 18;14(1):e0211053. doi: 10.1371/journal.pone.0211053 (PMC6338383; doi:10.1371/journal.pone.0211053)

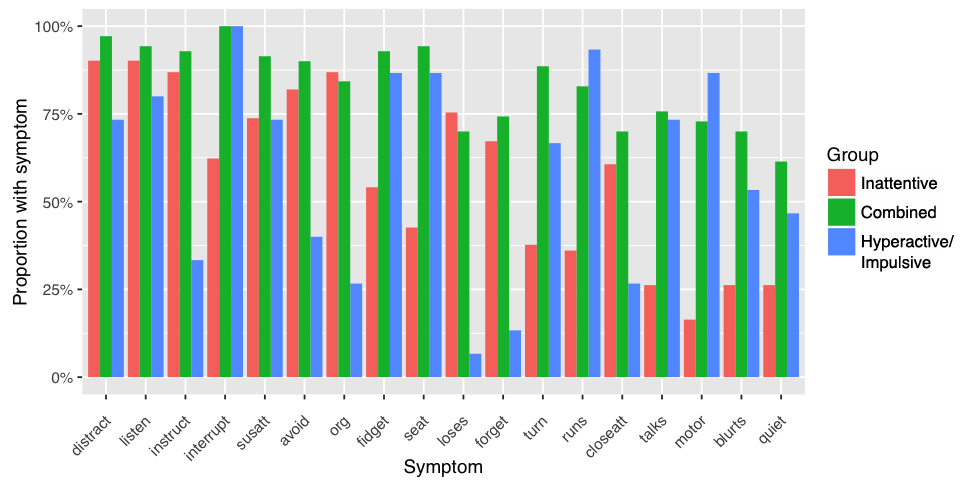

Supplement: S1 Fig — (TIF) [file pone.0211053.s001.tif]

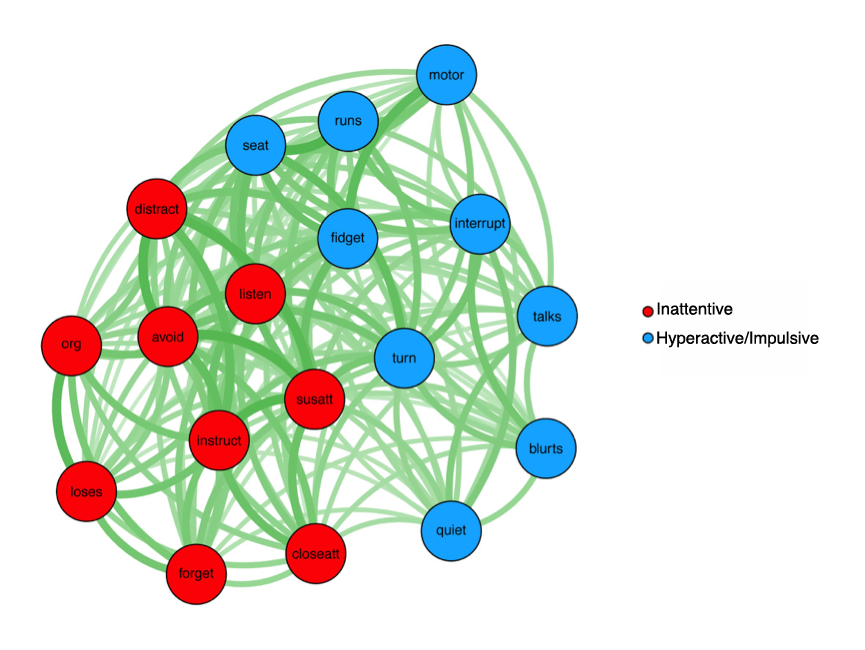

Supplement: S2 Fig — Nodes represent each of the 18 ADHD symptom criteria, connected by edges characterizing the zero-order Phi correlations between symptoms. Inattentive symptoms are presented in red and the hyperactive/impulsive symptoms are presented in blue. The green edges represent positive correlations and the thickness of the lines represent the magnitude of the association. (TIF) [file pone.0211053.s002.tif]
