# Supplementary material for: A network analysis approach to ADHD symptoms: More than the sum of its parts
Source: PLoS One. 2019 Jan 18;14(1):e0211053. doi: 10.1371/journal.pone.0211053 (PMC6338383; doi:10.1371/journal.pone.0211053)
Supplement: S1 Table — (DOCX) [file pone.0211053.s003.docx]

**S1 Table. Clinical and academic outcomes for children with ADHD.**

| ***Outcome*** |  |  | **ADHD**  ***n = 146*** | | **Control**  ***n = 209*** | |  |  |
| --- | --- | --- | --- | --- | --- | --- | --- | --- |
|  | Baseline | 3yr |  | n |  | n | T/χ2 | p  value |
| *Clinical measures* |  |  |  |  |  |  |  |  |
| Externalising comorbidity n(%) | • |  | 37 (25.3) | 146 | 10 (4.8) | 209 | 31.63 | <.001 |
| Internalising comorbidity n(%) | • |  | 75 (51.4) | 146 | 16 (7.7) | 209 | 86.16 | <.001 |
| Irritability, mean(sd) |  | • | 4.8 (3.3) | 109 | 1.6 (2.3) | 140 | 8.62 | <.001 |
| Social problems, mean(sd) | • |  | 3.0 (2.2) | 146 | 1.1 (1.3) | 199 | 9.31 | <.001 |
|  |  | • | 2.9 (2.4) | 113 | 1.0 (1.5) | 143 | 7.36 | <.001 |
| QoL emotional, mean(sd) |  | • | 54.4 (30.0) | 108 | 85.3 (18.2) | 139 | 9.44 | < .001 |
| QoL family, mean(sd) |  | • | 66.7 (23.9) | 108 | 90.2 (15.5) | 139 | 8.87 | < .001 |
| QoL time, mean(sd) |  | • | 74.1 (29.6) | 107 | 96.9 (10.8) | 138 | 3.01 | <.001 |
| *Academic cognitive functioning* |  |  |  |  |  |  |  |  |
| WRAT maths, mean(sd) | • |  | 91.3 (14.3) | 145 | 102.8 (13.5) | 209 | 7.61 | <.001 |
|  |  | • | 87.7 (14.5) | 114 | 99.1 (13.2) | 149 | 6.57 | <.001 |
| WRAT reading, mean(sd) | • |  | 98.1 (17.3) | 144 | 111.9 (13.6) | 209 | 8.02 | <.001 |
|  |  | • | 95.8 (14.4) | 113 | 106.4 (13.5) | 149 | 6.06 | <.001 |
| CELF language, mean(sd) | • |  | 15.5 (1.2) | 145 | 15.6 (1.1) | 209 | 0.80 | .43 |
|  |  | • | 18 (.6) | 114 | 18 (.5) | 149 | 0.00 | >.99 |
| Academic competence, mean(sd) | • |  | 86.2 (14.2) | 144 | 103.6 (11.9) | 205 | 12.03 | <.001 |
|  |  | • | 88.9 (13.8) | 98 | 103.5 (12.3) | 134 | 8.33 | <.001 |

CELF = Clinical Evaluation of Language Fundamentals; QoL= Quality of Life; WRAT = Wide Range Achievement Test.
